# Association between osmolality trajectories and mortality in patients with sepsis: a group-based trajectory model in large ICU open access databases

**DOI:** 10.3389/fmed.2025.1538322

**Published:** 2025-04-28

**Authors:** Yipeng Fang, Xuejun Shen, Aizhen Dou, Hui Xie, Keliang Xie

**Affiliations:** ^1^Department of Critical Care Medicine, Tianjin Medical University General Hospital, Tianjin, China; ^2^Department of Cardiology, The First Affiliated Hospital of Shantou University Medical College, Shantou, Guangdong, China; ^3^Fifth Clinical College, XinXiang Medical University, Xinxiang, Henan, China; ^4^Department of Anesthesiology, Tianjin Institute of Anesthesiology, Tianjin Medical University General Hospital, Tianjin, China

**Keywords:** osmolality, sepsis, in-hospital mortality, prognostic biomarker, group-based trajectory modeling

## Abstract

**Objective:**

The regulation of osmolality levels is controlled by the endocrine system, reflecting the body’s water and electrolyte balance. However, the relationship between dynamic osmolality trajectories and the prognosis of septic patients has not yet been reported. This study aims to investigate the predictive value of dynamic osmolality trajectories on mortality among patients with sepsis.

**Methods:**

A retrospective analysis was performed using the MIMIC IV and eICU-CRD databases. A total of 19,502 patients were included, 10,263 from MIMIC IV and 9,239 from eICU-CRD. Group-based trajectory modeling (GBTM) analysis was performed to identify distinct osmolality trajectories. The association between these trajectories and in-hospital mortality was assessed by logistic regression analysis and further adjusted for potential confounders. Subgroup analysis was used to identify potential interactive factors and to assess the robustness of the present findings.

**Results:**

Five distinct osmolality trajectories were identified. Patients in the persistent hyperosmolality trajectory (Trajectory-5) had significantly higher in-hospital mortality compared to other trajectories, with an increased risk of in-hospital mortality of 233% (OR 3.33, 95% CI 2.71–4.09) and 150% (OR 2.50, 95% CI 1.97–3.17) in MIMIC IV and eICU-CRD respectively, with Trajectory-2 as reference. A dynamic increase in osmolality (Trajectory-4) was also associated with a 68% (OR 1.68, 95% CI 1.39–2.03) and a 68% (OR 1.68, 95% CI 1.44–1.97) increase in the risk of death, compared with Trajectory-2. Conversely, maintaining osmolality in the range of 290–300 mOsm/L (Trajectory-1 and Trajectory-2) was associated with a lower risk of death. Our results remained stable in the IPWRA and subgroup analyses.

**Conclusion:**

Our findings suggest that dynamic changes in plasma osmolality are significantly associated with in-hospital mortality in septic patients. Osmolality trajectory model provides a potentially effective, easily accessible and cost-effective biomarker for the prognostic assessment and clinical management of sepsis.

## 1 Introduction

Plasma osmolality refers to the concentration of solute particles per unit mass of water, which is intricately regulated by the endocrine system, with key contributions from hormones such as antidiuretic hormone (ADH) and components of the renin-angiotensin-aldosterone system (RAAS) (1, 2). Osmolality is determined by the number of dissolved particles in a solution, primarily electrolytes (e.g., sodium, potassium) and non-electrolytes (e.g., glucose, urea). In biological systems, osmolality governs the movement of water across cell membranes via osmosis, a process critical for maintaining cellular volume and function. When extracellular osmolality increases, water moves out of cells, leading to cellular shrinkage; conversely, when extracellular osmolality decreases, water moves into cells, causing cellular swelling, both of which significantly induce injury and impair cellular function (3).

Abnormal osmolality, whether elevated (hyperosmolality) or reduced (hypo-osmolality), has been widely associated with increased mortality risk in a variety of diseases (4–7). Hyperosmolality, often observed in conditions such as hyperglycemia or dehydration, can lead to severe complications, including acute kidney injury and encephalopathy (8, 9). On the other hand, hypo-osmolality, commonly seen in hypoaldosteronism or cortisol deficiency, can cause significant morbidity and mortality due to cellular swelling and organ dysfunction (10). These adverse effects highlight the clinical importance of maintaining optimal osmolality levels.

Sepsis, defined by Sepsis 3.0 as infection-induced organ dysfunction, affects over 19 million patients annually, with 40% readmitted within 90 days (11–13). Abnormal osmolality in sepsis patients typically originates from multidimensional metabolic dysregulation, including glucose metabolism disorders, electrolyte homeostasis imbalance, and abnormal stress hormone secretion. Additionally, the endotoxin storm and inflammatory cytokine cascade during sepsis progression disrupt vascular endothelial integrity (14). Increased vascular permeability exacerbates osmolality imbalance through capillary leakage mechanisms, subsequently inducing tissue hypoperfusion and multi-organ dysfunction (15). As the central organ for osmoregulation, the kidney is particularly vulnerable to osmotic fluctuations. Hyperosmotic environments induce oxidative stress and cytoskeletal damage in renal tubular epithelial cells, significantly elevating the risk of acute kidney injury (16, 17). The pathophysiological linkage between osmolality changes and multi-organ injury aligns with sepsis-associated multiple organ dysfunction syndrome (MODS). Based on this, it is essential to focus on the impact of osmolality in sepsis patients. Research by Liang M et al. revealed a U-shaped pattern linking plasma osmolality to in-hospital mortality in septic cohort, indicating that both abnormal high and low osmolality are associated with poor clinical outcomes (4). Similarly, Shen Y et al. Also demonstrated this U-shaped relationship in critically ill patients (18). While single-time-point osmolality measurements has been widely reported as a prognostic biomarker in clinic, the dynamic trajectory may provide additional valuable information for predicting outcomes and guiding therapeutic interventions.

To date, no study has investigated the potential association between plasma osmolality trajectories and clinical outcomes, including in patients with sepsis. We hypothesize that osmolality trajectories are associated with in-hospital mortality in septic patients and that trends in osmolality changes may serve as a prognostic biomarker for sepsis. In the present study, we conducted a retrospective analysis using two publicly available critical care databases. Utilizing group-based trajectory model (GBTM) analysis, we aimed to examine the relationship between plasma osmolality trajectories and prognosis in septic patients.

## 2 Materials and methods

### 2.1 Data source and ethics approval

Present multicenter retrospective research utilizes data from two independent public intensive care databases: the Medical Information Mart for Intensive Care IV (MIMIC-IV) (19) and the eICU Collaborative Research Database (eICU-CRD) (20). The MIMIC-IV database is developed by the Computational Physiology Laboratory at the Massachusetts Institute of Technology (MIT) and includes clinical data from the Beth Israel Deaconess Medical Center (BIDMC). The eICU-CRD database encompasses over 200,000 critical care cases from multiple ICU centers across the United States, collected between 2014 and 2015. Author Yipeng Fang, obtaining access to these databases (ID: 43025968), was responsible for data extraction. Both the MIMIC-IV and eICU-CRD databases have received ethical approval from the MIT Institutional Review Board (20, 21). Informed consent was waived due to data anonymization and the fact that the analyses conducted do not influence clinical decision-making or patient care (20, 21). We structured our manuscript in accordance with the STROBE guidelines (22).

### 2.2 Population

#### 2.2.1 Inclusion criteria

(1) Adult patients (aged >18 years) diagnosed with sepsis during their ICU stay were screened for inclusion. Sepsis was diagnosed according to the Sepsis 3.0 definition (11). (2) Only the first admission records were analyzed for patients with multiple ICU admissions.

#### 2.2.2 Exclusion criteria

(1) Since the study required continuous seven-day osmolality data following a sepsis diagnosis, patients discharged or death within seven days were excluded. (2) Patients lacking osmolality data within the first 24 h of septic diagnosis.

### 2.3 Variable

Osmolality data within the first seven days following the sepsis diagnosis were used as the exposure variable. We collected daily results of serum sodium, potassium, glucose, and blood urea nitrogen (BUN) for each patient to calculate osmolality. When multiple results were available for a single day, only the first record was retained. The plasma osmolality was calculated using the following formula: Osmolality (mOsm/L) = 2 × [Na^+^(mmol/L) + K^+^(mmol/L)] + Glucose(mg/dL)/18 + BUN (mg/dL)/2.8 (23).

Clinical data, including demographic information (e.g., age, sex, body weight, race), comorbidities, laboratory results, disease severity scores, and specific treatments, were extracted from the MIMIC-IV and eICU-CRD databases utilizing PostgreSQL and PgAdmin4 software. Comorbidities were diagnosed using International Classification of Diseases (ICD) codes and the Charlson Comorbidity Index (details provided in Supplementary Table S1). We recorded the maximum values of white blood cell count, serum creatinine, and lactate, as well as the minimum values of platelet count and hemoglobin within 7 days of sepsis diagnosis. Disease severity was assessed using the maximum values of the Sequential Organ Failure Assessment (SOFA) score and the Acute Physiology Score (APS). Specific interventions were identified by the administration of diuretics, mechanical ventilation, and vasoactive therapy within 7 days of sepsis diagnosis. What’s more, the intake-output balance was analyzed during the 7-day period post-sepsis diagnosis.

### 2.4 Outcomes

The primary outcome was in-hospital mortality. Secondary outcomes included ICU mortality, length of hospital stay (hospital-LOS), and length of ICU stay (ICU-LOS). Additionally, in the MIMIC IV cohort, the differences in 28-day, 90-day and 1-year mortality rates across different trajectories were also detected.

### 2.5 Data clean

We excluded records without an osmolality result within the first 24 h of sepsis diagnosed. For osmolality missing values at other time points, we treated them as missing values and did not perform imputation (missing percentage of osmolality shown in Supplementary Table S2) (24). For normally distributed continuous variables, outliers were defined as values exceeding three standard deviations (SDs) from the mean. For skewed continuous variables, outliers were identified when values exceeding 1.5 times the interquartile range (IQR) from the 25th or 75th percentile. For outliers, we assigned a status of missing and handled them appropriately. When missing percentage of confounding factors was less than 10%, missing value was imputed by the mean or median according to their distribution. Regression imputation was performed for data with 10–20% missing values. Variables having more than 20% missing values were omitted from the analysis.

### 2.6 Statistical analysis

GBTM analysis was performed to investigate the dynamic change of osmolality within seven days of sepsis diagnosis to identify different plasma osmolality trajectories for grouping (25). The trajectories were conducted using the TRAJ procedure in STATA software according to the following standards. (1) The optimal number of trajectories was selected based on the Bayesian and Akaike information criteria (BIC and AIC) as well as the interpretability of the results (shown in Supplementary Table S3; Supplementary Figure S1); (2) all trajectory shapes were fitted using higher-order parameters (cubic), and only use lower-order parameters (linear or quadratic) for fitting when the fitting parameters do not have statistical significance; (3) the average posterior probability (AvePP) of each patient’s assignment to the corresponding osmolality trajectory was assessed, ensuring that the minimum acceptable threshold of 0.70 was met (24, 26).

Patients were divided into two groups according to the primary outcome and presented the baseline data between them. After identifying the trajectories, the outcomes across the different trajectories were compared. Continuous variables in normal distribution were presented as mean ± standard deviation (SD), while those with skewed distribution was shown as median with IQR. One-way ANOVA and Kruskal-Wallis tests were used for multigroup comparison appropriately. Counts and percentages were used to represent categorical variables, which were then analyzed through Chi-square tests.

To clarify the relationship between different trajectories and mortality, we used univariate and multivariate logistic regression analysis to validate our findings. In the multivariate analysis, a stepwise-backward approach was used for key variables selection (*p* < 0.05). In present study, age, race, body weight, diabetes, liver disease, chronic pulmonary disease, malignant cancer, lactic acid, hemoglobin, mechanical ventilation, vasoactive drugs, SOFA, and APSIII scores were considered as significant confounders by the stepwise-backward approach, and were included in the multivariate model. Variance inflation factor (VIF) was used to assess multicollinearity. VIF > 10 was taken as the evidence for multicollinearity. We transformed variables into binary variables to eliminate multicollinearity. What’s more, to eliminate the influence of confounders on our findings, we used Inverse Probability-Weighted Regression Adjustment (IPWRA) to balance the distribution of confounders. The IPWRA model included all significant confounders, which were identified by stepwise-backward approach in the multivariate analysis, and the estimated average treatment effect (ATE) was calculated in different osmolality trajectories, with trajectory-2 as the reference. Furthermore, subgroup analysis was performed according to the demographic information to further explore potential interactive factors and assess the robustness of the findings.

In present study, STATA (version 15.1 SE) software was used for statistical analysis, with statistical significance determined at a two-sided *p* value <0.05.

## 3 Results

### 3.1 Population selection and baseline information

**Figure 1:**
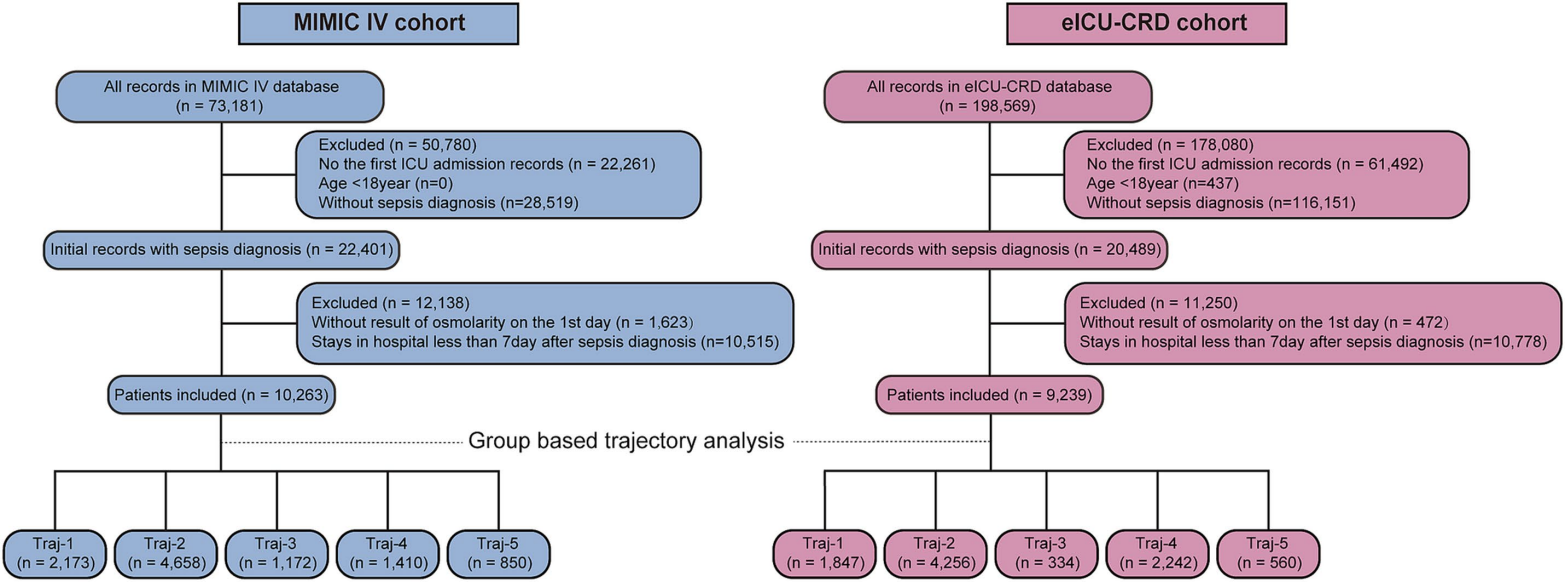
The flow chart of patient selection and trajectory.

Figure 1 presents the flow chart of present study. We obtained 10,263 and 9,239 eligible septic patients from the MIMIC and eICU-CRD databases, respectively. The MIMIC IV cohort included 9,015 survival and 1,248 non-survival patients. According to Table 1, non-survivors tended to be of older age, had a lower body weight, and a smaller proportion of white race (all *p* < 0.001), but there was no significant difference of sex (*p* = 0.149). More patients in the non-survival group had heart failure, atrial fibrillation, chronic pulmonary disease, chronic kidney disease, liver disease, and malignant cancer (all *p* < 0.05). The non-survival group had higher levels of white blood cells, lactate and creatinine, but decreased levels of serum platelets and hemoglobin (all *p* < 0.001). The plasma osmolality levels were higher in the non-survival group (all *p* < 0.001). A significantly higher cumulative fluid balance was observed in the non-survivor group during the 7 days following sepsis diagnosis (*p* < 0.001). What’s more, more patients in the non-survival group required mechanical ventilation, vasoactive drugs and diuretic intervention, and had higher SOFA and APSIII scores (all *p* < 0.05).

**Table 1:** Baseline information in alive and dead patients.

Variable	All patients		MIMIC IV database			eICU- CRD database		
	MIMIC IV	eICU	Alive	Death	*P* value	Alive	Death	*P* value
Number	10,263	9,239	9,015	1,248		8,011	1,228	
Age (years)	65.60 ± 16.45	65.60 ± 16.31	64.98 ± 16.58	70.09 ± 14.75	<0.001	65.14 ± 16.47	68.58 ± 14.86	<0.001
Male (%)	5,844 (56.94)	5,044 (54.59)	5,157 (57.20)	687 (55.05)	0.149	4,353 (54.34)	691 (56.27)	0.205
Ethnicity, white (%)	6,462 (62.96)	7,059 (76.40)	5,744 (63.72)	718 (57.53)	<0.001	6,128 (76.49)	931 (75.81)	0.601
Weight (kg)	83.03 ± 24.75	84.42 ± 28.57	83.32 ± 24.70	80.96 ± 25.02	0.002	84.82 ± 28.92	81.80 ± 26.00	<0.001
Comorbidity								
Coronary heart disease (%)	2,622 (25.55)	1,441 (15.60)	2,312 (25.65)	310 (24.84)	0.540	1,217 (15.19)	224 (18.24)	0.006
Heart failure (%)	3,064 (29.85)	1,667 (18.04)	2,633 (29.21)	431 (34.54)	<0.001	1,401 (17.49)	266 (21.66)	<0.001
Hypertension (%)	4,188 (40.81)	4,069 (44.04)	3,698 (41.02)	490 (39.26)	0.236	3,581 (44.70)	488 (39.74)	<0.001
Diabetes mellitus (%)	3,155 (30.74)	2,953 (31.96)	2,790 (30.95)	365 (29.25)	0.222	2,574 (32.13)	379 (30.86)	0.375
Atrial fibrillation (%)	3,450 (33.62)	1,232 (13.33)	2,924 (32.43)	526 (42.15)	<0.001	1,024 (12.78)	208 (16.94)	<0.001
Chronic pulmonary disease (%)	2,793 (27.21)	2,217 (24.00)	2,423 (26.88)	370 (29.65)	0.039	1,886 (23.54)	331 (26.95)	0.009
Chronic kidney disease (%)	2,269 (22.11)	1,250 (13.53)	1,953 (21.66)	316 (25.32)	0.004	1,076 (13.43)	174 (14.17)	0.481
Liver disease (%)	1,786 (17.40)	382 (4.13)	1,439 (15.96)	347 (27.80)	<0.001	296 (3.69)	86 (7.00)	<0.001
Malignant cancer (%)	1,577 (15.37)	1,418 (15.35)	1,299 (14.41)	278 (22.28)	<0.001	1,177 (14.69)	241 (19.63)	<0.001
Laboratory parameter								
White blood cell (k/uL)	15.0 (11.1,20.2)	15.4 (11.3,21.2)	14.7 (11.0,19.7)	17.5 (12.3,23.9)	<0.001	15.2 (11.1,20.8)	17.4 (12.6,23.5)	<0.001
Hemoglobin (g/dL)	8.69 ± 1.69	9.05 ± 1.88	8.75 ± 1.69	8.24 ± 1.61	<0.001	9.11 ± 1.88	8.68 ± 1.80	<0.001
Platelets (k/uL)	138 (86,201)	134 (85,189)	140 (92,199)	115 (54,180)	<0.001	143 (96,203)	121 (59,174)	<0.001
Creatinine (mg/dL)	1.3 (0.9,2.2)	1.5 (1.0,2.7)	1.2 (0.8,2.1)	1.7 (1.0,3.1)	<0.001	1.5 (0.9,2.7)	1.8 (1.1,3.1)	<0.001
Lactate (mmol/L)	1.9 (1.4,2.9)	2.1 (1.6,2.8)	1.9 (1.4,2.7)	2.5 (1.7,3.8)	<0.001	2.1 (1.6,2.7)	2.4 (1.7,3.4)	<0.001
Mean blood pressure (mmHg)	78.88 ± 9.79	79.62 ± 9.90	79.27 ± 9.82	76.04 ± 9.15	<0.001	80.01 ± 9.96	77.07 ± 9.12	<0.001
Osmolality (mmol/L)								
Initial value	303 (296,311)	304 (296,313)	302 (296,310)	306 (296,316)	<0.001	304 (296,313)	306 (297,316)	<0.001
Maximum value	311 (303,321)	313 (304,325)	310 (303,319)	318 (308,332)	<0.001	312 (304,323)	320 (310,331)	<0.001
Mean value	303 (296,311)	305 (298,314)	302 (296,310)	308 (298,319)	<0.001	305 (297,313)	310 (302,319)	<0.001
Minimum value	295 (289,302)	297 (20,305)	295 (289,301)	298 (289,308)	<0.001	297 (290,304)	300 (293,309)	<0.001
Intake and output balance (ml)	2,521 (−2,240,8,269)	−690 (−6,575,2,927)	2084 (−2,637,7,703)	5,577 (1,065,12,469)	<0.001	−761 (−6,655,2,706)	−299 (−6,045,4,392)	<0.001
Intervention								
Mechanical ventilation (%)	3,607 (35.15)	3,861 (41.79)	2,998 (33.26)	609 (48.80)	<0.001	3,180 (39.70)	681 (55.46)	<0.001
Vasoactive drug (%)	3,744 (36.48)	2,712 (29.35)	3,029 (33.60)	715 (57.29)	<0.001	2,214 (27.64)	498 (40.55)	<0.001
Diuretic exposure (%)	6,516 (63.49)	2,827 (30.60)	5,684 (63.05)	832 (66.67)	0.013	2,440 (30.46)	387 (31.51)	0.454
Disease severity score								
SOFA score	7 (5,10)	5 (4,7)	7 (5,9)	10 (7,14)	<0.001	5 (3,7)	6 (4,9)	<0.001
APSIII score	56 (42,75)	55 (42,71)	54 (41,71)	79 (62,101)	<0.001	54 (42,70)	63 (47,81)	<0.001

Tip: Continuous variables are displayed as mean (standard deviation) or median (first quartile–third quartile); categorical variables are displayed as count (percentage); APS, Acute Physiology Score, SOFA, Sequential Organ Failure Assessment.

The eICU-CRD cohort included 8,011 survival and 1,228 non-survival patients. Most of the differences between survival and non-survival patients were similar with the tendency in the MIMIC IV cohort. In the eICU-CRD cohort, there was no difference in the percentage of white race between the survival and non-survival groups (*p* = 0.601). The non-survival group had a higher proportion with coronary heart disease (all *p* < 0.001), and no significant difference was found in chronic kidney disease (*p* = 0.481). The trends in laboratory parameters and disease severity scores were consistent with the MIMIC IV cohort. Similarly, the plasma osmolality levels in the non-survival patients were also significantly higher than those in the survival patients (all *p* < 0.001). More patients in the non-survival group required mechanical ventilation and vasoactive treatment (all *p* < 0.001), but there was no difference in diuretic exposure (*p* = 0.454).

### 3.2 Osmolality trajectories

**Figure 2:**
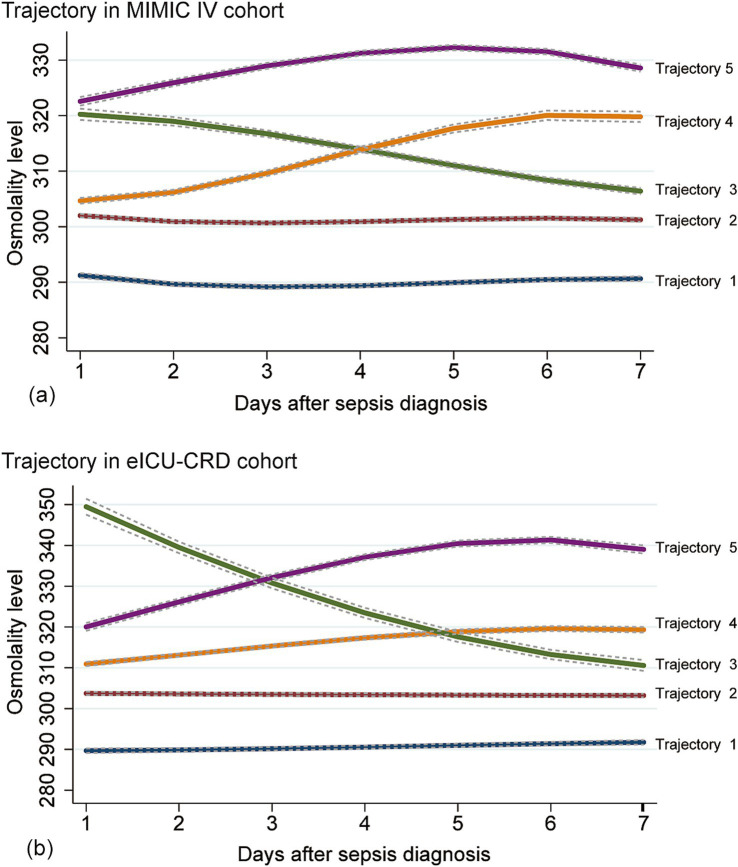
Osmolality-based trajectories in patients with sepsis. Upper panel: MIMIC IV cohort **(a)**; lower panel: eICU-CRD cohort **(b)**. Trajectory-1 (Lower Normality), an osmolality trajectory approximately 290 mOsm/L; Trajectory-2 (Upper Normality), an osmolality trajectory approximately 300 mOsm/L; Trajectory-3 (Abnormal-recovery), an osmolality trajectory higher than 310 mOsm/L and decreased gradually to 310 mOsm/L; Trajectory-4 (Normal-increase), an osmolality trajectory increased from normal range to the abnormal range; Trajectory-5 (Persistent Hyperosmolality), an osmolality trajectory higher than 310 mOsm/L constantly. Normal range of osmolality: 280–310 mOsm/L.

We fitted two to eight osmolality trajectories for each cohort, and the details of BIC/AIC values and the visualized results were shown in Supplementary Table S3 and Supplementary Figure S1. As the number of trajectories increased, the BIC and AIC values gradually increased. The maximum of BIC and AIC appeared when eight trajectories were fitted. When the number of trajectories exceeded five, there were significant differences in the tendency of trajectories between the two cohorts, further complicating their clinical interpretation. An excessive number of trajectories can also result in an excessively low lowest proportion per class, thereby compromising the quality of the trajectory model. Finally, we chose the model with five trajectories for further analysis (shown in Figure 2). In the MIMIC cohort (Figure 2a), five trajectories included 2,173, 4,658, 1,172, 1,410, and 850 patients, respectively. In the eICU-CRD cohort (Figure 2b), the trajectories included 1,847, 4,256, 334, 2,242, 560 patients, respectively (see in Figure 1; Table 2).

**Table 2:** The details of different trajectories.

Trajectory	Naming	Meaning	Number (%)	AvePP
Trajectory-1	Stable Low-Normal	Within the reference range, approximately 290 mOsm/L	2,173 (21.17%), 1,847 (19.99%)	0.92, 0.91
Trajectory-2	Stable High-Normal	Within the reference range, approximately 300 mOsm/L	4,658 (45.39%), 4,256 (46.07%)	0.92, 0.88
Trajectory-3	Recovery Pattern	Decline from abnormal to normal levels	1,172 (11.42%), 334 (3.62%)	0.88, 0.93
Trajectory-4	Deterioration Pattern	Rise from normal to abnormal levels	1,410 (13.74%), 2,242 (24.27%)	0.88, 0.89
Trajectory-5	Persistent Hyperosmolality	Persistent high-abnormal level, over 310 mOsm/L constantly	850 (8.28%), 560 (6.06%)	0.95, 0.93

### 3.3 Comparisons of osmolality and outcomes among osmolality trajectories

As shown in Table 3, in the MIMIC IV cohort, patients in trajectory-5 had the highest in-hospital mortality rate (27.88%), followed by trajectory-4, trajectory-3, trajectory-1, and trajectory-2. All mortality indicators had consistent tendency, that trajectory-5 having the highest mortality and trajectory-2 the lowest. Additionally, there were significant differences in hospital-LOS and ICU-LOS among the trajectories (all *p* < 0.001).

**Table 3:** Comparison of osmolarity levels and outcomes among different trajectories.

Variable	Trajectory-1	Trajectory-2	Trajectory-3	Trajectory-4	Trajectory-5	*P* value
MIMIC IV cohort						
Number	2,173	4,658	1,172	1,410	850	
Osmolality (Osmo/L)	291 (288,293)	301 (298,304)	313 (310,317)	312 (310,316)	327 (324,332)	<0.001
2*Sodium (mmol/L)	271 (267,274)	278 (275,281)	285 (280,289)	284 (279,288)	290 (285,296)	<0.001
2*Potassium (mmol/L)	7.9 (7.4,8.3)	7.9 (7.5,8.4)	8.0 (7.5,8.5)	8.0 (7.6,8.5)	8.1 (7.6,8.7)	<0.001
Glucose (mg/dL)	6.4 (5.8,7.2)	6.8 (6.0,7.9)	7.4 (6.3,9.0)	7.6 (6.6,9.2)	8.4 (7.1,10.4)	<0.001
BUN/2.8 (mg/dL)	5.0 (3.5,7.0)	7.2 (5.0,10.3)	12.1 (8.6,17.6)	12.9 (9.0,17.6)	20.8 (15.3,27.4)	<0.001
Hospital mortality (%)	208 (9.57)	416 (8.93)	150 (12.80)	237 (16.81)	237 (27.88)	<0.001
ICU mortality (%)	115 (5.29)	251 (5.39)	90 (7.68)	145 (10.28)	145 (17.06)	<0.001
28-day mortality	249 (11.46)	504 (10.82)	190 (16.21)	275 (19.50)	287 (33.76)	<0.001
90-day mortality	437 (20.11)	833 (17.88)	315 (26.88)	412 (29.22)	383 (45.06)	<0.001
1-year mortality	638 (29.36)	1,258 (27.01)	462 (39.42)	549 (38.94)	478 (56.24)	<0.001
Hospital LOS (days)	14 (10,21)	14 (10,21)	15 (11,23)	16 (12,25)	16 (12,25)	<0.001
ICU LOS (days)	4 (2,8)	5 (3,10)	6 (3,11)	8 (5,14)	9 (5,15)	<0.001
eICU-CRD cohort						
Number	1,847	4,256	334	2,242	560	
Osmolality (Osmo/L)	292 (288,294)	303 (300,307)	325 (320,332)	316 (313,320)	330 (328,335)	<0.001
2*Sodium (mmol/L)	270 (265,274)	279 (275,283)	292 (287,298)	285 (280,290)	292 (286,296)	<0.001
2*Potassium (mmol/L)	7.7 (7.4,8.2)	7.8 (7.3,8.3)	7.8 (7.3,8.3)	7.9 (7.4,8.5)	8.0 (7.5,8.8)	<0.001
Glucose (mg/dL)	6.5 (7.5,7.6)	7.1 (6.1,8.6)	8.2 (8.6,10.4)	8.1 (6.8,10.0)	9.2 (7.6,10.9)	<0.001
BUN/2.8 (mg/dL)	5.5 (3.8,7.9)	8.4 (5.9,12.0)	17.4 (12.3,22.8)	14.2 (10.5,19.2)	22.7 (17.8,28.1)	<0.001
Hospital mortality (%)	141 (7.63)	471 (11.07)	41 (12.28)	417 (18.60)	158 (28.21)	<0.001
ICU mortality (%)	56 (3.03)	201 (4.72)	22 (6.59)	182 (8.12)	73 (13.04)	<0.001
Hospital LOS (days)	12 (9,18)	13 (10,18)	13 (9,18)	15 (10,21)	15 (10,22)	<0.001
ICU LOS (days)	4 (2,7)	5 (2,8)	4 (2,8)	7 (4,11)	8 (4,12)	<0.001

Tip: Continuous variables were displayed as mean (standard deviation) or median (first quartile–third quartile); categorical variables were displayed as count (percentage); ICU, intensive care unit; LOS, length of stay.

Similar to the MIMIC IV cohort, patients in trajectory-5 had the highest in-hospital mortality (28.21%), followed by trajectory-4 and trajectory-3 in the eICU-CRD cohort. However, unlike what we found in the MIMIC IV cohort, the in-hospital mortality of trajectory-1 (7.63%) was lower than that of trajectory-2 (11.07%). There were also significant differences in hospital-LOS and ICU-LOS among the different trajectories in the eICU-CRD cohort (all *p* < 0.001). The baseline information between different trajectories of MIMIC IV and eICU-CRD cohorts was shown in Supplementary Tables S4, S5.

**Figure 3:**
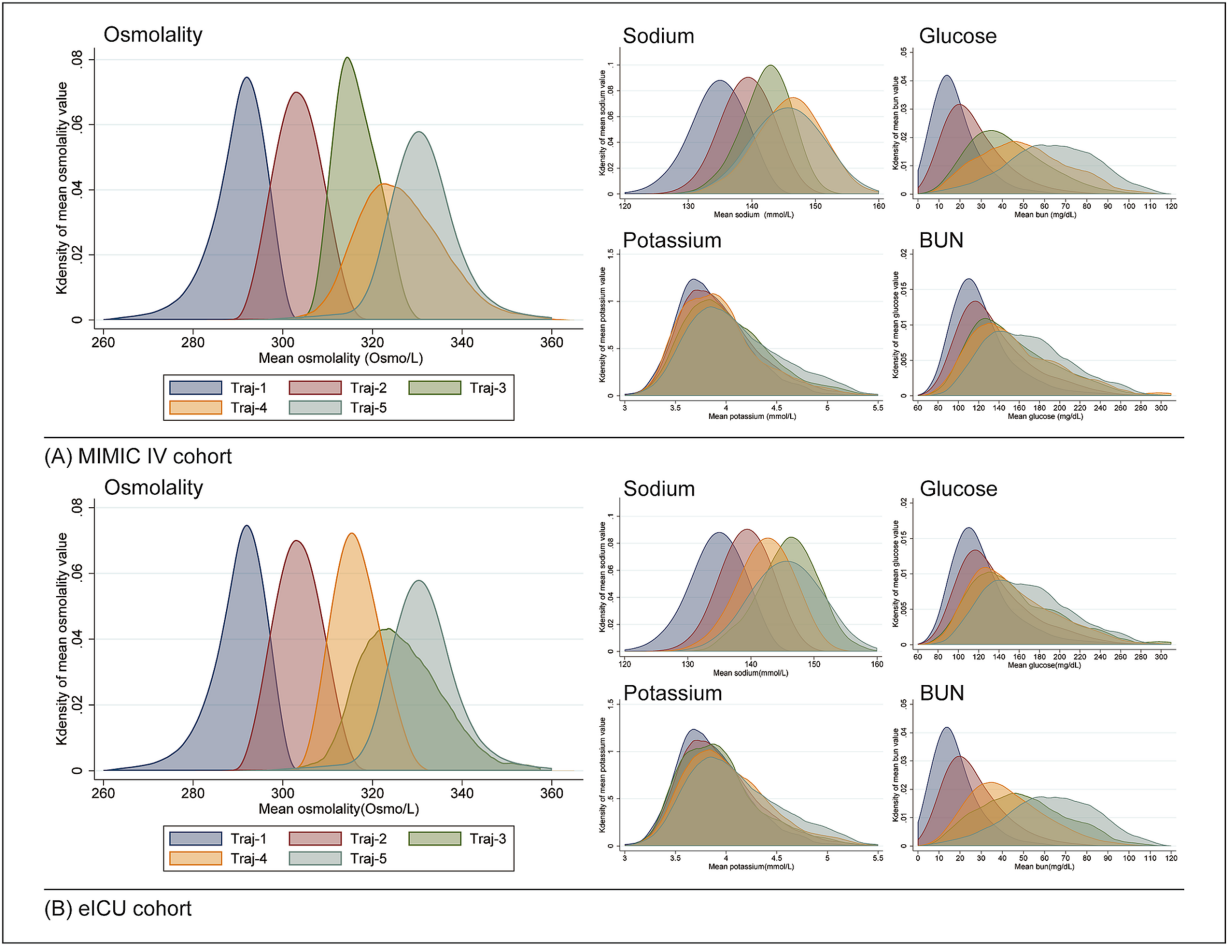
Distribution of osmolality and related indicators across different trajectories using kernel density curves. **(A)** MIMIC-IV cohort; **(B)** eICU cohort.

Table 3 displays the distribution of sodium, potassium, glucose, and BUN levels for each trajectory in both cohorts. Kernel density curves (see in Figure 3) further illustrate the distribution patterns of osmolality and these indicators across trajectories. Notably, all indicators showed trends that were fully aligned with osmolality, highlighting their consistent relationship.

### 3.4 Logistic regression analysis

The association between osmolality trajectories and in-hospital mortality was further investigated by logistic regression analysis (shown in Table 4). With trajectory-2 as the reference in the MIMIC-IV cohort, the crude risk of in-hospital morality was 1.49 times for trajectory-3, 2.06 times for trajectory-4, and 3.94 times for trajectory-5, respectively. No significant difference was found between trajectory-2 and trajectory-1 (*p* = 0.392). After adjusting confounding factors, both trajectory-1 and trajectory-3 had no significant difference compared with trajectory-2 (*p* = 0.251 and 0.122).

**Table 4:** Logistic regression analysis between different trajectories and the risk of in-hospital death.

Trajectory	MIMIC IV cohort				eICU-CRD cohort			
	Unadjusted model		Adjusted model		Unadjusted model		Adjusted model	
	OR (95%)	*P* value	OR (95%)	*P* value	OR (95%)	*P* value	OR (95%)	*P* value
Trajectory-1	1.08 (0.91–1.29)	0.392	1.12 (0.92–1.35)	0.251	0.66 (0.54–0.81)	<0.001	0.76 (0.61–0.94)	0.011
Trajectory-2	Reference		Reference		Reference		Reference	
Trajectory-3	1.49 (1.23–1.83)	<0.001	1.19 (0.96–1.47)	0.122	1.12 (0.80–1.58)	0.499	1.11 (0.77–1.61)	0.577
Trajectory-4	2.06 (1.73–2.45)	<0.001	1.68 (1.39–2.03)	<0.001	1.84 (1.60–2.12)	<0.001	1.68 (1.44–1.97)	<0.001
Trajectory-5	3.94 (3.29–4.72)	<0.001	3.33 (2.71–4.09)	<0.001	3.16 (2.57–3.89)	<0.001	2.50 (1.97–3.17)	<0.001

Tip: Variables included in the adjusted model were selected through a stepwise backward elimination process. The final model incorporated age, race, weight, diabetes, heart failure, liver disease, malignant cancer, lactate, white blood cell count, creatinine, mean arterial pressure, mechanical ventilation, vasoactive drug use, diuretic use, Intake and output balance, SOFA score, and APSIII score. APS, Acute Physiology Score, SOFA, Sequential Organ Failure Assessment.

Notably, compared with trajectory-2 in the eICU-CRD cohort, patients in the trajectory-1 had a significantly decreased risk of in-hospital mortality (*p* < 0.001), but no in trajectory-3 (*p* = 0.499). The tendency of trajectory-4 and trajectory-5 was similar with what we found in the MIMIC IV cohort. After adjusting confounding factors, patients in trajectory-4 and trajectory-5 still had significantly higher risk of in-hospital mortality with trajectory-2 as reference (all *p* < 0.001), but no statistically significant difference between the risk of the trajectory-2 and trajectory-3 (*p* = 0.577). Patients in the trajectory-1 still had a lower risk of in-hospital mortality (*p* = 0.011) in the adjusted model.

In the IPWRA analysis (see in Supplementary Table S6), the average increased in-hospital mortality attributable to the osmolality trajectory relative to trajectory 2 was 5.1 and 6.7% for trajectory 4, 19.4 and 14.9% for trajectory 5 in both cohorts (all *p* < 0.001). Not significant ATE was found for trajectory 3 (all *p* > 0.05). In MIMIC IV cohort, trajectory 1 was associated with a 1.9% increase in in-hospital mortality (ATE 0.019, *p* = 0.039) while it was associated with a 1.7% reduced in-hospital mortality in the eICU-CRD cohort (ATE −0.017, *p* = 0.093).

### 3.5 Subgroup analysis

**Figure 4:**
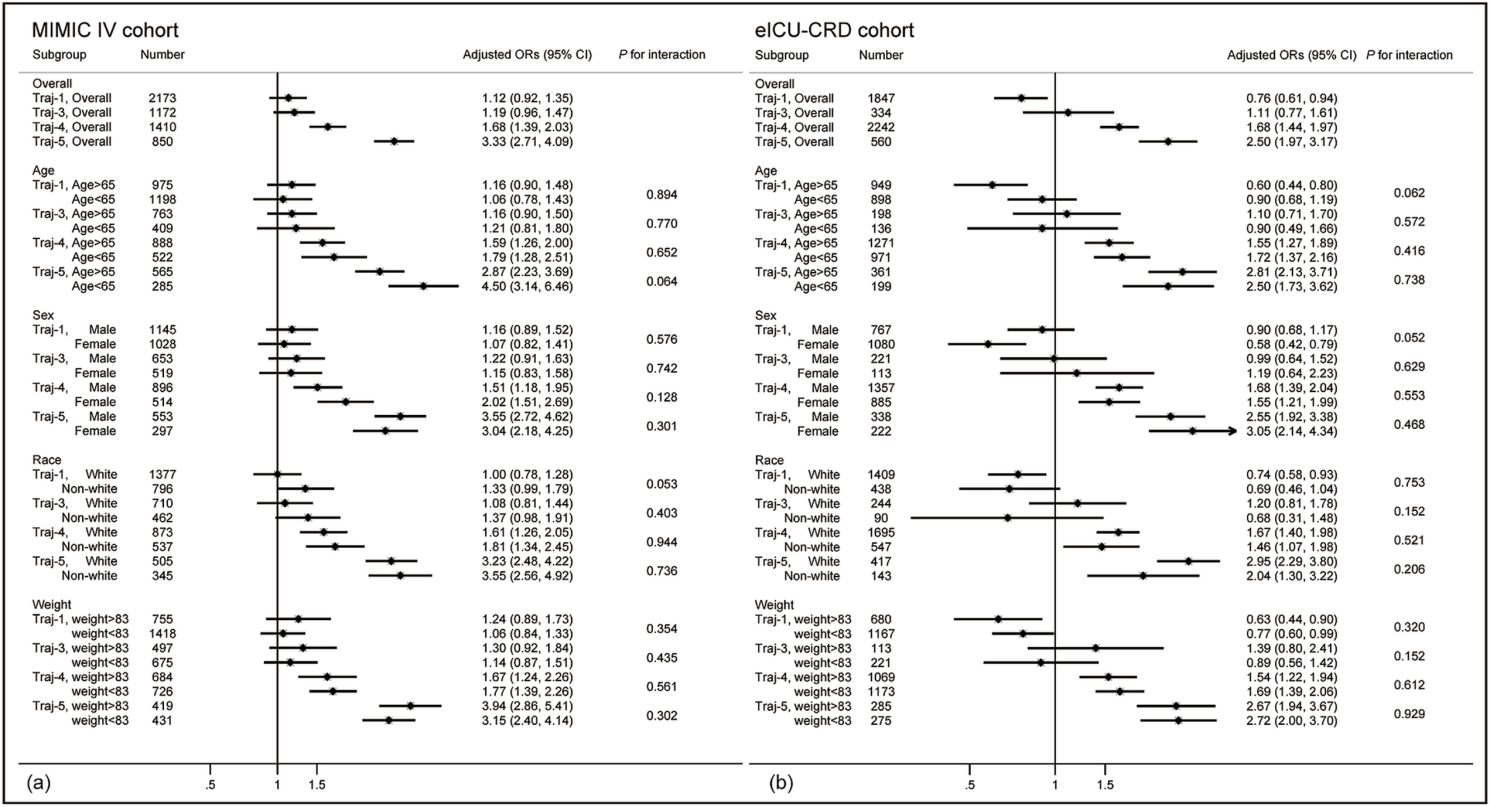
Forest plot of subgroup analysis. **(a)** Forest plot in the MIMIC IV cohort. **(b)** Forest plot in the eICU-CRD cohort. Results are all referenced to trajectory-2. Blue represents trajectory-1, green represents trajectory-3, orange represents trajectory-4, and purple represents trajectory-5. The analysis was conducted using the multivariable logistic regression model from Table 3.

We conducted a subgroup analysis of patients based on demographic information (see in Figure 4). In both two cohorts (see in Figure 4a), no significant interactions were found in both cohorts (all *P* for interaction > 0.05). The trends of present subgroup analysis were consistent with the overall cohort. For trajectory-1, all subgroups in the MIMIC IV cohort indicated higher risk of in-hospital mortality (OR>1), while the eICU-CRD cohort (shown in Figure 4b) suggested lower risk of in-hospital mortality (OR<1), with trajectory-2 as reference. All subgroups supported that patient in the trajectory-4 and trajectory-5 had higher risk of in-hospital mortality (OR>1). For trajectory-3, no significant statistical differences were observed in any subgroups.

### 3.6 Combined analysis

We conducted a combined analysis of two databases, including a total of 19,502 patients, and reconstructed the trajectories. The trajectories and trends observed in the combined cohort were consistent with those from the independent analyses of MIMIC-IV and eICU-CRD (see Supplementary Figure S2; Supplementary Table S7). We found that Trajectory-1 was associated with the lowest mortality, followed by Trajectory-2, although no statistically significant difference was observed after adjusting for confounders (shown in Table 5). Trajectory-5 was linked to the highest mortality risk, followed by Trajectory-4 and Trajectory-3, which aligns with the results from the independent MIMIC-IV and eICU-CRD analyses.

**Table 5:** Predictive value of different osmolality trajectories on the risk of in-hospital death in the combined cohort using logistic regression analysis.

Trajectory	Unadjusted model		Adjusted model	
	OR (95%)	*P* value	OR (95%)	*P* value
Trajectory-1	0.84 (0.74–0.95)	0.005	0.93 (0.81–1.07)	0.307
Trajectory-2	Reference		Reference	
Trajectory-3	1.44 (1.22–1.71)	<0.001	1.26 (1.05–1.51)	0.012
Trajectory-4	2.03 (1.82–2.27)	<0.001	1.79 (1.58–2.01)	<0.001
Trajectory-5	3.66 (3.18–4.21)	<0.001	3.10 (2.65–3.64)	<0.001

Tip: Variables included in the adjusted model were selected through a stepwise backward elimination process. The final model incorporated age, race, weight, diabetes, heart failure, liver disease, malignant cancer, lactate, white blood cell count, creatinine, mean arterial pressure, mechanical ventilation, vasoactive drug use, diuretic use, Intake and output balance, SOFA score, and APSIII score. APS, Acute Physiology Score, SOFA, Sequential Organ Failure Assessment.

## 4 Discussion

This is a multi-center retrospective study analyzing relationship between mortality and the trajectory model based on osmolality in patients with sepsis. Compared to previous studies, we used longitudinal osmolality data rather than a single osmolality value to predict the clinical outcomes in the septic cohorts. The main findings of this study indicate that persistently abnormal high osmolality and novel developed hyperosmolality is associated with increased risk of in-hospital mortality in patients with sepsis. Maintaining osmolality levels within the normal range is associated with better prognosis; however, this study has not reached a unified conclusion regarding whether stable levels of 290 mOsm/L and 300 mOsm/L have different impacts on the outcomes. After eliminating the effects of potential confounders, these findings were still robust in both multivariable logistic regression and the IPWRA analysis.

Plasma osmolality is a useful and easily obtained marker in clinic, reflecting the homeostasis of endocrine system, and the balance of sodium, glucose and BUN in the body. Osmolality abnormalities are common and closely associated with electrolyte disturbances, glucose imbalances, and renal dysfunction. Abnormal osmolality changes have been reported to be an independent risk factor for poor prognosis in various diseases (8, 9, 27). There is a U-shaped pattern linking plasma osmolality to in-hospital death, indicating that both excessively high and low osmolality changes are detrimental for patients with sepsis (4). The main limitation of previous studies is their focus on the osmolality value at a single point in time, which does not reflect the impact of dynamic changes in osmolality, which is a dramatically fluctuating indicator over time. Present study categorized patients into five trajectories based on longitudinal osmolality data, effectively addressing the above shortcoming. Among all trajectories, osmolality exhibited different dynamic changing patterns. Stable and normal osmolality levels (trajectory-1 and trajectory-2) were associated with the lowest risk of death in patients with sepsis. This finding is consistent with previous studies, emphasizing the importance of maintaining osmolality levels within the normal range (4, 23). Our study established two osmolality trajectories belonging to the normal range, the 290 mOsm/L and 300 mOsm/L trajectories (trajectory-1 and trajectory-2). However, the relationship between osmolality and mortality for Trajectory-1 and Trajectory-2 remains inconsistent across both cohorts. Maintaining osmolality steadily within the range of 290–300 mOsm/L may be beneficial for patients with sepsis. Notably, baseline imbalances in critical prognostic factors, like heart failure, inflammatory markers, renal dysfunction, lactate, mechanical ventilation, and SOFA scores, between trajectory groups may introduce confounding in our results. Future studies with additional data are needed to explore whether lower osmolality levels are associated with reduced mortality risk and to determine the optimal reference range for osmolality.

The detrimental effects of hyperosmolality are widely recognized in clinical practice. In our study, no significant difference was found in term of mortality between trajectory-2 and Trajectory-3; however, patients in Trajectory-5, representing those with persistently abnormal hyperosmolality, exhibited a significantly increased risk of mortality. A study found the highest quintile of plasma osmolality (Q5 > 303.21) increased the risk of 28-day mortality in septic patients by 99% (HR 1.99, 95% CI 1.74–2.28) (4). Additionally, Heng et al. reported that 34.9% of patients with septic shock exhibits hyperosmolality, which is associated with a higher risk of mortality by 47% (OR 1.470, 95% CI 1.140–1.895) (23). Elevated osmolality levels are also closely linked to the risk of AKI (8). The negative effect of hyperosmolality is widely reported, as it has been associated with poor outcomes also in those with acute ischemic stroke (5), heart failure (6) and diabetes (7). The detrimental effects of elevated osmolality on disease prognosis arise through several interconnected mechanisms. Firstly, hyperosmolality disrupts fluid and electrolyte homeostasis, causing osmotic diuresis, dehydration, and cellular shrinkage. This impairs organelle function, particularly in mitochondria and the nucleus, leading to energy deficits, activation of apoptosis, and accelerated cell death (7, 28, 29). Secondly, hyperosmolality alters hemodynamics by increasing blood viscosity and promoting a hypercoagulable state, primarily through elevated blood glucose levels that inhibit fibrinolysis. These changes reduce cellular perfusion, exacerbate tissue hypoxia, and impair brain function (5, 30). Thirdly, hyperosmolality triggers intracellular stress responses, including calcium overload, excessive ROS production, and endoplasmic reticulum stress, which collectively contribute to cellular apoptosis and systemic inflammation (31, 32).

Another important finding of the present study is that dynamic increases in osmolality, even in patients with initially normal osmolality, were also associated with an increased risk of death (trajectory-4). This finding emphasizes the importance of dynamic monitoring of osmolality. Using single time points as exposure variables, as in previous studies, may overlook this specific population and lead to poor clinical outcomes due to neglect. Changes in osmolality are closely related to fluid management, especially the intake of solutions and the regulation of blood glucose, both of which play significant roles in osmolality regulation. In clinical practice, osmolality levels are often used to guide fluid management; however, there are no established guidelines regarding the optimal osmolality levels that may improve the prognosis of septic patients. The findings of this study provide important clues for future research on osmolality interventions. Due to the limitations of the retrospective study design, we cannot establish a causal relationship solely based on the observed association between osmolality levels and mortality. Future studies could explore whether actively controlling osmolality within an appropriate range (possibly 290–300 mOsm/L) can improve patient outcomes, which represents a promising direction for further investigation. What’s more, for patients with initially normal osmolality, avoiding abnormal fluctuations in osmolality levels is also crucial.

Regrettably, trajectories obtained in present study did not present the population with hypo-osmolality. However, this pathological phenomenon should not be overlooked. Previous studies have shown that compared to the patients with osmolality levels of 291.38–296.29 mmol/L, the osmolality of ≤285.80 mmol/L is independently associated with a 59% increased risk of mortality in septic patients (HR 1.59, 95% CI 1.39–1.83) (4). In septic patients with shock, hypo-osmolality increases the risk of mortality by 30% (OR 1.301, 95% CI 1.075–1.575) (23). Furthermore, compared to hyperosmolality (OR 1.198, 95% CI 1.199–1.479), the presence of hypo-osmolality is a stronger independent risk factor for the development of acute kidney injury (AKI) (OR 1.332, 95% CI 1.199–1.479) (8). The effects of hypo-osmolality on tissue and cell damage are primarily mediated through cellular deformation and swelling due to water influx. Additionally, the presence of hyponatremia would deplete the adaptive response to hypo-osmotic stress for cells and tissues (33). With a reduction in intracellular organic osmolytes, cells become more susceptible to damage caused by increased glutamine and the subsequent hyperosmotic effect of water entering the cells, leading to cellular and tissue injury (33).

Our study has several advantages. We utilized two independent public databases to validate our hypothesis, which allowed us to include a great number of participants. This substantial sample size enhances the credibility and reliability of our findings. As a multicenter retrospective study, our results have greater generalizability and representativeness. The combined cohort analysis yielded consistent results with those from the individual cohorts, further attesting to the universal applicability of the osmolality trajectories across different patient populations. To our knowledge, we are the first clinical study to examine osmolality trajectories, providing a potentially effective, easily accessible, and cost-efficient biomarker for the prognostic evaluation of sepsis.

The present study also has several limitations and therefore the conclusions should be interpreted with caution. As a retrospective study, potential confounding factors due to the inherent limitations of the study design may affect the accuracy of our findings. Although we used multivariate logistic regression and IPWRA to minimize confounding and validate our results in two independent databases to increase its reliability, potential biases remain existed. The use of different types of intravenous fluids and diuretics could significantly influence osmolality levels. However, detailed information on these factors was not systematically collected in this study, potentially impacting the accuracy of the findings. In addition, as most of the data were derived from an American population, the generalizability of these findings to other countries and regions requires further validation. Furthermore, the low proportion of hypo-osmolality in our cohort limits the ability of our osmolality trajectories to further evaluate the impact of hypo-osmolality in septic patients. What’s more, the osmolality in this study was calculated using the formula rather than directly measured, which represents a significant limitation, particularly in septic patients who often present with renal dysfunction, hypoalbuminemia, and increased unmeasured anions. Finally, although our findings are based on longitudinal osmolality data, the observational nature of the present study precludes the establishment of causal relationships, highlighting the need for further high-quality clinical research to confirm our conclusions.

## 5 Conclusion

Present study elucidates the association between distinct osmolality trajectories and clinical outcomes in patients with sepsis. The osmolality trajectory model offers a novel approach for guiding clinical management of sepsis and represents a potential new biomarker for prognosis and treatment decisions.

## Data Availability

Publicly available datasets were analyzed in this study. This data can be found at: MIMIC-IV (Version 2.2, accessible at: https://physionet.org/content/mimiciv/2.2/) and eICU-CRD databases (Version 2.0, accessible at: https://physionet.org/content/eicu-crd/2.0).
