# Supplementary material for: Association between osmolality trajectories and mortality in patients with sepsis: a group-based trajectory model in large ICU open access databases
Source: Front Med (Lausanne). 2025 Apr 28;12:1538322. doi: 10.3389/fmed.2025.1538322 (PMC12066632; doi:10.3389/fmed.2025.1538322)
Supplement: Supplementary file 1 [file Data_Sheet_1.DOCX]

**Association between osmolality trajectories and mortality in patients with sepsis a group-based trajectory model in large ICU open access databases**

**Supplemental materials list**

**Table S1** Details for comorbidity definition

**Table S2** Distribution of missing values for osmolality

**Table S3** The parameter of different trajectory model using GBTM analysis

**Table S4** Baseline information between different trajectories in MIMIC IV cohort

**Table S5** Baseline information between different trajectories in eICU-CRD cohort

**Table S6** The average treatment effects of different osmolality trajectory on hospital mortality in multivariable IPWA analysis

**Table S7** Different osmolality trajectories and outcomes in combined cohort

**Fig. S1** The graph of different trajectory model using GBTM analysis

**Fig. S2** Osmolality-based trajectories in the combined cohort.

**Table S1** The ICD codes used to screen for complications

| Complications | ICD codes |
| --- | --- |
| Diabetes mellitus | 25000, 24900, 24901, 24911, 24920, 24940, 24941, 24950, 24951, 24960, 24961, 24970, 24971, 24980, 24981, 24990, 24991, 25001, 25002, 25003, 25010, 25011, 25012, 25013, 25020, 25021, 25022, 25023, 25030, 25031, 25032, 25033, 25040, 25041, 25042, 25043, 25050, 25051, 25052, 25053, 25060, 25061, 25062, 25063, 25070, 25071, 25072, 25073, 25080, 25081, 25082, 25083, 25090, 25091, 25092, 25093, E0800, E0810, E0821, E0822, E08319, E083513, E0840, E0842, E0843, E0851, E08649, E0865, E089, E0900, E0910, E0921, E0922, E09319, E0940, E0942, E0943, E0951, E0952, E09621, E09649, E0965, E098, E099, E1010, E1011, E1021, E1022, E1029, E10311, E10319, E10321, E103213, E103219, E10329, E103291, E103292, E103293, E103299, E103312, E103313, E103319, E10339, E103391, E103393, E103399, E103411, E103413, E10349, E103491, E10351, E103511, E103512, E103513, E103519, E103522, E103523, E103531, E103532, E103559, E10359, E103591, E103592, E103593, E103599, E1036, E1039, E1040, E1041, E1042, E1043, E1044, E1049, E1051, E1052, E1059, E10610, E10618, E10620, E10621, E10622, E10628, E10641, E10649, E1065, E1069, E108, E109, E1100, E1101, E1110, E1121, E1122, E1129, E11311, E11319, E11321, E113211, E113212, E113213, E113219, E11329, E113291, E113292, E113293, E113299, E11331, E113311, E113313, E113319, E11339, E113391, E113392, E113393, E113399, E11341, E113413, E113419, E11349, E113491, E113492, E113493, E113499, E11351, E113511, E113512, E113513, E113519, E113521, E113522, E113532, E113542, E113553, E11359, E113591, E113592, E113593, E113599, E1136, E1137X9, E1139, E1140, E1141, E1142, E1143, E1144, E1149, E1151, E1152, E1159, E11610, E11618, E11620, E11621, E11622, E11628, E11641, E11649, E1165, E1169, E118, E119, E1300, E1310, E1311, E1321, E1322, E1329, E13319, E1340, E1342, E1343, E1351, E1359, E13621, E13622, E13649, E1365, E1369, E138, E139 |
| Hypertension | 4019, 4011, I10 |
| Coronary heart disease | 4111, 4142, 41181, 41402, 41404, 41405, 41407, I2101, I2102, I2109, I2111, I2119, I2121, I240, I2510, I25110, I25111, I25118, I25119, I25700, I25708, I25709, I25710, I25711, I25718, I25719, I25720, I25721, I25728, I25729, I25758, I25790, I25810, I25811, I2582, T82213A, T82218A, T82855A, T82855D, T82855S, V4581, V4582, Z951, Z955, Z9861 |
| Acute or chronic heart failure | 4280, 4281, 4289, 39891, 40201, 40211, 40291, 40401, 40403, 40411, 40413, 40491, 40493, 42820, 42821, 42822, 42823, 42830, 42831, 42832, 42833, 42840, 42841, 42842, 42843, I0981, I110, I130, I132, I5020, I5021, I5022, I5023, I5030, I5031, I5032, I5033, I5040, I5041, I5042, I5043, I50810, I50811, I50812, I50813, I50814, I5082, I5083, I5084, I5089, I509, I97130, I97131 |
| Chronic kidney disease | 5851, 5852, 5853, 5854, 5855, 5859, 28521, 40300, 40301, 40310, 40311, 40390, 40391, 40400, 40401, 40403, 40410, 40411, 40413, 40490, 40491, 40492, 40493, D631, E0822, E0922, E1022, E1122, E1322, I120, I129, I130, I1310, I1311, I132, N181, N182, N183, N184, N185, N189 |
| Atrial fibrillation | 42731, I480, I481, I4811, I4819, I482, I4820, I4821, I4891 |
| Cerebral infarction | I63132, I63133, I63139, I6319, I6320, I63211, I63212, I63213, I63219, I6322, I63231, I63232, I63233, I63239, I6329, I6330, I63311, I63312, I63313, I63321, I63322, I63331, I63332, I63333, I63341, I63342, I63343, I63349, I6339, I6340, I63411, I63412, I63413, I63419, I63421, I63422, I63423, I63429, I63431, I63432, I63433, I63439, I63441, I63442, I63443, I63449, I6349, I6350, I63511, I63512, I63513, I63521, I63522, I63523, I63529, I63531, I63532, I63533, I63539, I63541, I63542, I63543, I63549, I6359, I636, I638, I6381, I6389, I639, I6930, I6931, I69310, I69311, I69312, I69313, I69315, I69318, I69319, I69320, I69321, I69322, I69323, I69328, I69331, I69333, I69334, I69341, I69344, I69351, I69352, I69353, I69354, I69359, I69364, I69365, I69369, I69390, I69391, I69392, I69393, I69398, V1254, Z8673 |
| Cerebral hemorrhage | 431, I619, I610, I611, I612, I613, I614, I615, I616, I618, I6911, I69110, I69111, I69112, I69118, I69119, I69120, I69121, I69122, I69128, I69131, I69132, I69134, I69141, I69144, I69151, I69152, I69153, I69154, I69159, I69164, I69165, I69169, I69190, I69191, I69192, I69193, I69198 |
| Gastrointestinal bleeding | 4560, 45620, 53021, I8501, I8511, K2211, K2901, K2921, K2941, K2951, K2961, K2971, K2981, K2991, K31811, K5701, K5711, K5713, K5721, K5731, K5733, K5751, K5791, K5793, |
| Anemia | 2819, 2800, 2801, 2808, 2809, 2810, 2811, 2812, 2813, 2823, 2828, 2829, 2830, 2839, 2849, 2850, 2851, 2853, 2858, 28310, 28319, 28409, 28489, D461, D4620, D4621, D4622, D464, D500, D508, D509, D510, D511, D513, D518, D519, D520, D521, D528, D529, D531, D538, D539, D588, D589, D590, D591, D592, D594, D598, D599, D6109, D611, D612, D613, D6189, D619, D62, D641, D643, D644, D6489, D75A, O368230 |
| Sepsis | According to the Sepsis 3 structured view |
| Chronic pulmonary disease | According to the Charlson structured view |
| Liver disease | According to the Charlson structured view |
| Malignant cancer | According to the Charlson structured view |

**Table S2** Distribution of missing values for osmolality

| **MIMICIV** | **Sodium** | **Potassium** | **Glucose** | **BUN** | **Osmolality (%)** |
| --- | --- | --- | --- | --- | --- |
| Day1 | NA | NA | NA | NA | NA |
| Day2 | 167 | 204 | 219 | 192 | 288 (2.8) |
| Day3 | 265 | 310 | 356 | 316 | 473 (4.6) |
| Day4 | 375 | 410 | 463 | 429 | 574 (5.6) |
| Day5 | 511 | 540 | 657 | 576 | 799 (7.8) |
| Day6 | 697 | 712 | 844 | 770 | 978 (9.5) |
| Day7 | 914 | 917 | 1,068 | 1,099 | 1,217 (11.9) |
| **eICU** |  |  |  |  |  |
| Day1 | NA | NA | NA | NA | NA |
| Day2 | 721 | 683 | 433 | 752 | 787 (8.5) |
| Day3 | 1,046 | 993 | 693 | 1,079 | 1,102 (11.9) |
| Day4 | 1,482 | 1,400 | 1,041 | 1,520 | 1,546 (16.7) |
| Day5 | 1,875 | 1,806 | 1,346 | 1,912 | 1,950 (21.1) |
| Day6 | 2,232 | 2,162 | 1,666 | 2,250 | 2,284 (24.7) |
| Day7 | 2,605 | 2,515 | 1,981 | 2,629 | 2,662 (28.8) |

**Table S3** The parameter of different trajectory model using GBTM analysis

|  | MIMIC IV | | | | eICU-CRD | | | |
| --- | --- | --- | --- | --- | --- | --- | --- | --- |
| Trajectories | BIC | AIC | Lowest PPC | Lowest AvePP | BIC | AIC | Lowest PPC | Lowest AvePP |
| 2 | -256274.18 | -256238.00 | 30.8% | 95.7% | -214857.90 | -214822.24 | 32.9% | 94.0% |
| 3 | -250264.79 | -250210.52 | 15.8% | 93.2% | -210625.29 | -210571.81 | 13.5% | 91.0% |
| 4 | -247634.76 | -247562.40 | 10.0% | 90.3% | -208697.61 | -208626.30 | 6.8% | 88.0% |
| 5 | -245486.92 | -245396.47 | 8.2% | 88.2% | -206619.18 | -206530.04 | 3.6% | 88.2% |
| 6 | -243737.85 | -243629.31 | 5.0% | 89.1% | -205227.12 | -205120.15 | 1.7% | 84.8% |
| 7 | -242671.07 | -242544.44 | 4.1% | 82.0% | -204183.81 | -204059.02 | 1.3% | 83.7% |
| 8 | -241738.01 | -241593.29 | 3.7% | 84.3% | -203271.32 | -203128.69 | 1.3% | 83.7% |

Tip: PPC, Proportions per class%; AvePP, Average posterior probability

**Table S4** Baseline information between different trajectories in MIMIC IV cohort

| **Variable** | **Trajectory 1** | **Trajectory 2** | **Trajectory 3** | **Trajectory 4** | **Trajectory 5** | **P-value** |
| --- | --- | --- | --- | --- | --- | --- |
| Number | 2,173 | 4,658 | 1,172 | 1,410 | 850 |  |
| Age (years) | 61.17±17.22 | 64.96±16.38 | 69.28±15.34 | 68.65±15.20 | 70.28±15.05 | <0.001 |
| Male (%) | 1,145 (52.69) | 2,597(55.75) | 653 (63.55) | 896(63.55) | 553(65.06) | <0.001 |
| Ethnicity, white (%) | 1,377(63.37) | 2,997(64.34) | 710(60.58) | 873(61.91) | 505(59.41) | 0.017 |
| Weight (kg) | 78.41±22.26 | 83.69±25.67 | 82.73±24.42 | 86.30±23.78 | 86.25±25.99 | <0.001 |
| Comorbidity |  |  |  |  |  |  |
| Coronary heart disease (%) | 442(20.34) | 1,230(26.41) | 306(26.11) | 416(29.50) | 228(26.82) | <0.001 |
| Heart failure (%) | 449(20.66) | 1,323(28.40) | 433(36.95) | 485(34.40) | 374(44.00) | <0.001 |
| Hypertension (%) | 906(41.69) | 2,012(43.19) | 435(37.12) | 554(39.29) | 281(33.06) | <0.001 |
| Diabetes mellitus (%) | 423(19.47) | 1,372(29.45) | 453(38.65) | 517(36.67) | 390(45.88) | <0.001 |
| Atrial fibrillation (%) | 592(27.24) | 1,535(32.95) | 415(35.41) | 540(38.30) | 368(43.29) | <0.001 |
| Chronic pulmonary disease (%) | 524(24.11) | 1,280(27.48) | 349(29.78) | 404(28.65) | 236(27.76) | 0.003 |
| Chronic kidney disease (%) | 214(9.85) | 873(18.74) | 408(34.81) | 414(29.36) | 360(42.35) | <0.001 |
| Liver disease (%) | 426(19.60) | 705(15.14) | 211(18.00) | 266(18.87) | 178(20.94) | <0.001 |
| Malignant cancer (%) | 390(17.95) | 696(14.94) | 172(14.68) | 202(14.33) | 117(13.76) | 0.005 |
| Laboratory parameter |  |  |  |  |  |  |
| White blood cell (k/uL) | 14.5(10.5,19.5) | 14.7(11.0,19.7) | 15.3(11.4,20.9) | 15.8(11.9,21.5) | 16.1(12.1-21.8) | <0.001 |
| Hemoglobin (g/dL) | 8.67±1.67 | 8.81±1.69 | 8.50±1.68 | 8.63±1.69 | 8.37±1.64 | <0.001 |
| Platelets (k/uL) | 143(89,220) | 141(91,201) | 137(83,199) | 124(75,180) | 132(75,189) | <0.001 |
| Creatinine (mg/dL) | 0.9(0.7,1.3) | 1.2(0.8,1.8) | 1.8(1.1,3.2) | 1.8(1.2,3.0) | 2.5(1.6,4.0) | <0.001 |
| Lactate (mmol/L) | 1.8(1.3,2.7) | 1.9(1.4,2.8) | 2.0(1.4,2.9) | 2.1(1.5,3.3) | 2.1(11.5,3.1) | <0.001 |
| Mean blood pressure (mmHg) | 78.20±10.00 | 78.97±9.82 | 78.86±10.02 | 79.31±9.09 | 79.47±9.87 | 0.002 |
| Intake and output balance(ml) | 2612(-2690,9312) | 2398(-2336,8334) | 3530(-1632,9621) | 2085(-2155,6805) | 2428(-2118,7190) | <0.001 |
| Intervention |  |  |  |  |  |  |
| Mechanical ventilation (%) | 594(27.34) | 1625(34.89) | 365(31.14) | 654(46.38) | 369(43.41) | <0.001 |
| Vasoactive drug (%) | 672(30.92) | 1,670(35.85) | 405(34.56) | 661(46.888) | 336(39.53) | <0.001 |
| Diuretic exposure (%) | 1,150(52.92) | 2,922(62.73) | 724(61.77) | 1,109(78.65) | 611(71.88) | <0.001 |
| Disease severity score |  |  |  |  |  |  |
| SOFA score | 6(4,8) | 7(5,9) | 8(5,11) | 9(6,12) | 9(7,12) | <0.001 |
| APSIII score | 48(36,64) | 53(40,71) | 64(50,81) | 65(50,85) | 72(57,89) | <0.001 |

Tip: Continuous variables are displayed as mean (standard deviation) or median (first quartile–third quartile); categorical variables are displayed as count (percentage); APS, Acute Physiology Score, SOFA, Sequential Organ Failure Assessment.

**Table S5** Baseline information between different trajectories in eICU-CRD cohort

| **Variable** | **Trajectory 1** | **Trajectory 2** | **Trajectory 3** | **Trajectory 4** | **Trajectory 5** | **P-value** |
| --- | --- | --- | --- | --- | --- | --- |
| Number | 1,847 | 4,256 | 334 | 2,242 | 560 |  |
| Age (years) | 61.05±17.32 | 65.35±16.23 | 71.01±14.61 | 68.32±15.04 | 68.43±15.52 | <0.001 |
| Male (%) | 949(51.38) | 2,265(53.22) | 198(59.28) | 1,271(56.69) | 361(64.46) | <0.001 |
| Ethnicity, white (%) | 1,409(76.29) | 3,294(77.40) | 244(73.05) | 1,695(75.60) | 417(74.46) | 0.172 |
| Weight (kg) | 80.48±26.39 | 84.37±28.86 | 79.71±27.21 | 87.42±29.62 | 88.60±27.75 | <0.001 |
| Comorbidity |  |  |  |  |  |  |
| Coronary heart disease (%) | 224(12.13) | 644(15.13) | 69(20.66) | 399(17.80) | 105(18.75) | <0.001 |
| Heart failure (%) | 225(12.18) | 745(17.50) | 66(19.76) | 483(21.54) | 148(26.43) | <0.001 |
| Hypertension (%) | 710(38.44) | 1,861(43.73) | 169(50.60) | 1,062(47.37) | 267(47.68) | <0.001 |
| Diabetes mellitus (%) | 397(21.49) | 1,347(31.65) | 137(41.02) | 830(37.02) | 242(43.21) | <0.001 |
| Atrial fibrillation (%) | 187(10.12) | 582(13.67) | 43(12.87) | 332(14.81) | 88(15.71) | <0.001 |
| Chronic pulmonary disease (%) | 375(20.30) | 1,024(24.06) | 70(20.96) | 601(26.81) | 147(26.25) | <0.001 |
| Chronic kidney disease (%) | 164(8.88) | 532(12.50) | 60(17.96) | 392(17.48) | 102(18.21) | <0.001 |
| Liver disease (%) | 93(5.04) | 154(3.62) | 4(1.20) | 98(4.37) | 33(5.89) | 0.001 |
| Malignant cancer (%) | 308(16.68) | 634(14.90) | 41(12.28) | 360(16.06) | 75(13.39) | 0.087 |
| Laboratory parameter |  |  |  |  |  |  |
| White blood cell (k/uL) | 14.9(10.7,20.5) | 15.1(11.0,20.7) | 15.7(11.9,21.4) | 16.2(11.9,22.0) | 18.0(18.9,23.8) | <0.001 |
| Hemoglobin (g/dL) | 9.1±1.8 | 9.2±1.9 | 8.6±1.8 | 8.9±1.9 | 8.7±1.8 | <0.001 |
| Platelets (k/uL) | 146(95,212) | 144(98,203) | 128(84,176) | 134(85,187) | 123(73,180) | <0.001 |
| Creatinine (mg/dL) | 1.0(0.7,1.6) | 1.4(0.9,2.4) | 2.6(1.7,4.5) | 2.0(1.3,3.3) | 2.8(2.0,4.0) | <0.001 |
| Lactic acid (mmol/L) | 2.2(1.6,2.5) | 2.1(1.6,2.7) | 2.2(1.6,2.8) | 2.2(1.7,3.0) | 2.4(1.7,3.2) | <0.001 |
| Mean blood pressure (mmHg) | 79.58±10.34 | 79.43±9.76 | 79.17±10.43 | 79.93±9.73 | 80.29±9.84 | 0.135 |
| Intake and output balance(ml) | -859(-6659,2472) | -675(-6541,2721) | -140(-6270,4432) | 790(-6692,3338) | -213(-6284,4468) | 0.009 |
| Intervention |  |  |  |  |  |  |
| Mechanical ventilation (%) | 559(30.27) | 1,719(40.39) | 119(35.63) | 1,155(51.52) | 309(55.18) | <0.001 |
| Vasoactive drug (%) | 435(23.55) | 1,249(29.35) | 88(26.35) | 75(33.23) | 195(34.82) | <0.001 |
| Diuretic exposure (%) | 532(28.80) | 1,239(29.11) | 65(19.46) | 799(35.64) | 192(34.29) | <0.001 |
| Disease severity score |  |  |  |  |  |  |
| SOFA score | 4(3,6) | 5(3,7) | 6(4,8) | 6(4,8) | 6(5,9) | <0.001 |
| APSIII score | 48(37,62) | 53(41,68) | 66(53,82) | 61(48,78) | 65(51,83) | <0.001 |

Tip: Continuous variables are displayed as mean (standard deviation) or median (first quartile–third quartile); categorical variables are displayed as count (percentage); APS, Acute Physiology Score, SOFA, Sequential Organ Failure Assessment.

**Table S6** The average treatment effects of different osmolality trajectory on hospital mortality in multivariable IPWA analysis

|  | MIMIC IV cohort | | eICU-CRD cohort | |
| --- | --- | --- | --- | --- |
| Variable | Adjusted ATE (95% CI) | *P* value | Adjusted ATE (95% CI) | *P* value |
| Trajectory-1 | 0.019(0.001-0.037) | 0.039 | -0.017(-0.037- 0.003) | 0.093 |
| Trajectory-2 | Reference | | Reference | |
| Trajectory-3 | 0.013(-0.006-0.032) | 0.187 | 0.032(-0.028- 0.093) | 0.297 |
| Trajectory-4 | 0.051(0.029-0.072) | <0.001 | 0.067 (0.047-0.087) | <0.001 |
| Trajectory-5 | 0.194(0.1510-0.237) | <0.001 | 0.149 (0.094-0.205) | <0.001 |

ATE: average treatment effect; CI: confidence interval

**Table S7** Different osmolality trajectories and outcomes in combined cohort

|  | **Trajectory-1** | **Trajectory-2** | **Trajectory-3** | **Trajectory-4** | **Trajectory-5** | ***P* value** |
| --- | --- | --- | --- | --- | --- | --- |
| **Combined cohort** |  |  |  |  |  |  |
| Number | 4,531 | 8,916 | 1,353 | 3,460 | 1,242 |  |
| Hospital mortality (%) | 388(8.56) | 898(10.07) | 188(13.90) | 641(18.53) | 361(29.07) | <0.001 |
| ICU mortality (%) | 195(4.30) | 455(5.10) | 98(7.24) | 330(9.54) | 202(16.26) | <0.001 |
| Hospital LOS (days) | 13(10,20) | 13(10,20) | 14(10,21) | 16(11,23) | 16(11,23) | <0.001 |
| ICU LOS (days) | 4(2,8) | 5(2,9) | 5(3,10) | 8(4,13) | 9(4,13) | <0.001 |

Tip: Continuous variables were displayed as mean (standard deviation) or median (first quartile–third quartile); categorical variables were displayed as count (percentage); ICU, intensive care unit; LOS, length of stay.


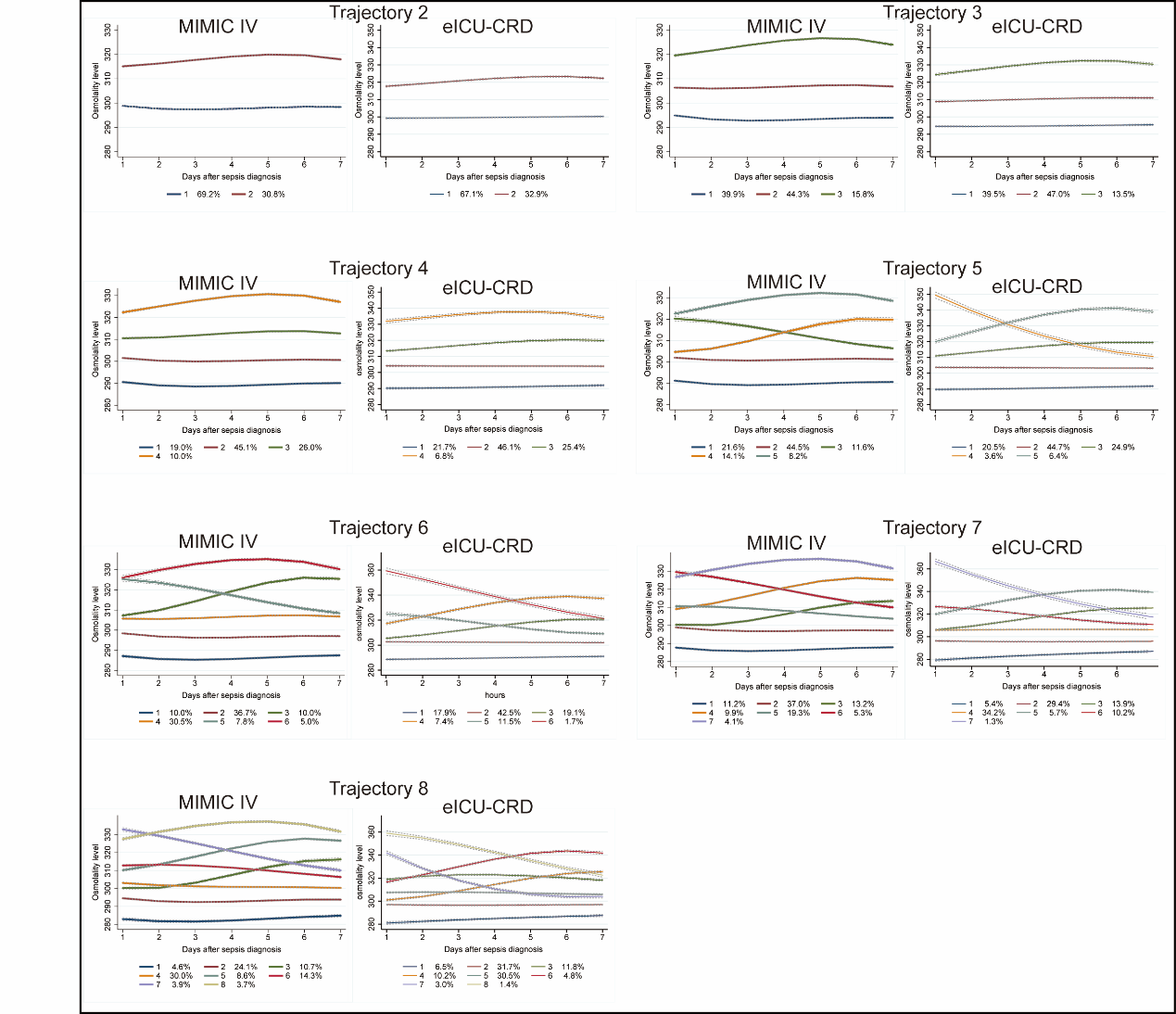


**Fig. S1** The graph of different trajectory model using GBTM analysis


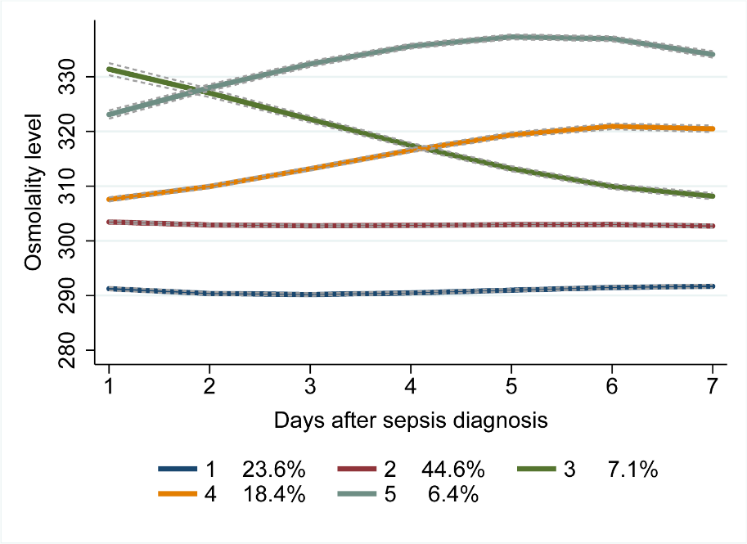


**Fig. S2** Osmolality-based trajectories in the combined cohort.
